# Human microbiome and microbiota identification for preventing and controlling healthcare-associated infections: A systematic review

**DOI:** 10.3389/fpubh.2022.989496

**Published:** 2022-12-01

**Authors:** Pamela Tozzo, Arianna Delicati, Luciana Caenazzo

**Affiliations:** ^1^Legal Medicine Unit, Laboratory of Forensic Genetics, Department of Cardiac, Thoracic, Vascular Sciences and Public Health, University of Padova, Padova, Italy; ^2^Department of Pharmaceutical and Pharmacological Sciences, University of Padova, Padova, Italy

**Keywords:** human microbiome, human microbiota, healthcare-associated infections (HAI), prevention, infections control measures, risk factors, hospitalization, systematic review

## Abstract

**Objective:**

This systematic review describes the role of the human microbiome and microbiota in healthcare-associated infections (HAIs). Studies on the microbiota of patients, healthcare environment (HE), medical equipment, or healthcare workers (HCW) and how it could be transmitted among the different subjects will be described in order to define alarming risk factors for HAIs spreading and to identify strategies for HAIs control or prevention.

**Methods:**

This review was performed in adherence to the Preferred Reporting Items for Systematic Reviews and Meta-Analyses (PRISMA) guidelines. After retrieval in databases, identification, and screening of available records, 36 published studies were considered eligible and included in the review.

**Results:**

A multifaceted approach is required and the analyses of the many factors related to human microbiota, which can influence HAIs onset, could be of paramount importance in their prevention and control. In this review, we will focus mainly on the localization, transmission, and prevention of ESKAPE (*Enterococcus faecium, Staphylococcus aureus, Klebsiella pneumoniae, Acinetobacter baumannii, Pseudomonas aeruginosa*, and *Enterobacter species*) bacteria and Clostridium difficile which are the most common pathogens causing HAIs.

**Conclusions:**

Healthcare workers' microbiota, patient's microbiota, environmental and medical equipment microbiota, ecosystem characteristics, ways of transmission, cleaning strategies, and the microbial resistome should be taken into account for future studies on more effective preventive and therapeutic strategies against HAIs.

## Introduction

Healthcare-Associated Infections (HAIs) Are one of the major threats to hospitalized patients and a major public health burden. HAIs are nosocomial-acquired infections that are not present or incubating in the patient on their hospitalization but they should manifest at least 48 h after admission to the hospital (1–3). HAIs are considered those infections acquired in any healthcare facilities such as hospitals, nursing homes, ambulatory, rehabilitation centers, and any other facilities, both public or private, which provide healthcare or diagnostic service to individuals (4). HAIs have become one of the major challenges for the healthcare services of western countries due to an aging society and the increased level of immunocompromised patients in healthcare facilities, in particular for those in intensive care units (ICUs) (1, 5–8). HAIs incidence increase with prolonged hospitalization and with the utilization of invasive life-prolonging procedures including venous and arterial catheterizations, tracheal intubation, urinary catheterization, invasive intracranial pressure monitoring, and placement of sterile site drainage catheters (1, 5, 6, 9). Moreover, HAIs represent one of the most frequent complications of hospitalization worldwide, with an annual incidence ranging approximately from 5 to 15% of all hospitalized inpatients. Consequently, increasing attention has been given to HAIs by government health institutions (for instance the European Centre for Disease Prevention and Control and the Centre for Disease Control) which have implemented specific surveillance programs to collect data and have issued regulations for the mandatory reporting of such infections. In Europe, every year, more than 4 million people developed HAIs, with 16 million (6%) additional hospital days and ~37.000 deaths. In Italy, specifically, between 450 and 700 thousand people are affected by HAIs every year. According to a 2013 national prevalence study, the prevalence of patients with at least one HAI is 6,3% (1, 10–15).

Given the clinical impact and the costs associated with HAIs, current research in this field is aimed to develop protocols for HAIs prevention or management and among the different possible solutions the first suggested was a more accurate hygiene protocol (1, 2, 15). Everything started in 1846, with Ignaz Semmelweis and his contributions in terms of hand washing, so much so that since then hand hygiene has been proposed multiple times as an important solution to control the spread of HAIs (10, 16). Nevertheless, proper hands washing is observed in < 40% of cases–even in units with critically ill patients–due to poor bath-room placement, lack of time, forgetfulness or rejection of the usual recommendations, or negligence (17–19). Therefore, since HAIs arise from complex systems influenced by many factors, it is needed a more pluralistic approach for proper infection control, which cannot be limited to hand washing but should involve a multidisciplinary team including hospital doctors, infection control nurses, microbiologists, architects, and engineers with expertise in building design and facilities management.

Among the factors which influence the development of HAIs, there are the biological characteristics of the infectious agents involved as well as the susceptibility of the host to both exogenous and endogenous microorganisms (5, 20, 21). The human body harbors trillions of microbes that form a diverse ecosystem including bacteria, viruses, fungi, and protozoa; in particular, collectively they are named “human microbiota” and their genomes are referred to as the “human microbiome” (22–26). Previous studies had argued that, within the human body, microbial cells outnumber human cells 10-fold (27, 28), but recent research has demonstrated that the microbial cells are abundant as the human ones, with a more realistic ratio of about 1.3 between the former and the latter (29). The human microbiome plays an essential role in health, lipid metabolism, colonization resistance to transient organisms, and immune response (30–32) and can be influenced by different factors such as body location (33, 34), diet (35), sex (36, 37), ethnicity (38), and age (38, 39). In addition, the microbial community can also be shaped by habits (40), relationships (41, 42), disease state (43, 44), and environment (42, 45). With the latter a bidirectional influence exists; indeed, on the one hand, the environment can influence the microbiota of people who live there, on the other hand, humans release their bacteria into the surrounding environment, changing its microbial composition (27). Moreover, the Human Microbiome Project and other studies on the human microbiome have revealed a wide diversity in composition and abundance of the microbiome within an individual (alpha diversity) with differences that appear consistent between individuals (beta diversity) (33, 34, 46). In terms of human microbiome complexity, the increased number of studies on the human microbiome and the huge contribution to defining the role of the microbiome in health and diseases allowed us to highlight the direct/indirect mechanisms of action with which the microbiota act to confer protection against pathogens (7). Once pathogens entered the organism, in addition to the protection conferred by the microbiota, antimicrobial therapies could be useful were it not for the increased number of antimicrobial-resistant (AMR) microorganisms (47, 48). Nowadays, despite gram-negative bacteria remain still being associated with HAIs, gram-positive bacteria (such as *Enterococci* and *Staphylococcus epidermidis*) have become most frequently associated with HAIs in the context of both surgical site or bloodstream infection (1, 48). However, despite all these changes and the identification of the high number of possible factors (such as host genetics, age, nutrition, and environment) which can influence the human microbiota and its role in health and disease, it is poorly understood what “healthy microbiota” really means (24, 46). Therefore, this systematic review aims to analyze in detail how variations in the microbiome can be associated with HAIs. In particular, studies on the microbiota of patients, healthcare environment (HE), medical equipment, or healthcare workers (HCW) and how it could be transmitted among the different subjects will be analyzed in order to define alarming factors for HAIs spreading and to identify strategies for HAIs control or prevention.

## Methods

### Research parameters

This systematic review was performed in adherence to the Preferred Reporting Items for Systematic Reviews and Meta-Analyses (PRISMA) guidelines (49). A systematic literature review regarding HAIs, the related microbiota, and methods for HAIs control and prevention was conducted using public electronic databases (PubMed and Scopus).

The works were selected according to the query: ((((“Healthcare-associated infection”) OR (“Healthcare-associated infections”)) AND (microbiome)) OR (((“nosocomial infection”) OR (“nosocomial infections”)) AND (microbiome))) AND ((control) OR (prevention)). One of the reviewers (A.D.) carried out the initial search of the papers and the consensus of research supervisors (L.C. and P.T.) was required.

### Inclusion and exclusion criteria

Inclusion criteria were as follows: (1) English language; (2) Date of publication, i.e., articles published from 2000 to 2022; (3) Availability of both abstract and full text; and 4. only articles dealing with the role of the microbiome in the prevention of HAIs in healthcare facilities settings.

Papers have been excluded applying the following exclusion criteria: (A) systemic reviews or any other works (for instance chapter of a book) which is not experimental or which did not analyse the relationship among HAIs and the microbiota of different sources such as HCW, patients, HE or healthcare instrumentation (HI – medical equipment); (B) articles which are focussed on fungi or virus and not on bacteria; (C) articles which explained how HAIs are treated highlighting the emergence of new drugs; (D) articles which considered HAIs without an overview of the microbiota correlated; (E) articles which analyzed the microbiota of patients but in infections or other diseases which are not HAIs or; (F) articles which included content not relevant to the aim of the review.

### Research workflow

A total of 272 works were identified through database searching. An English language filter was applied to start the screening process and narrow the search to 256 works. Duplicates (91 works) were removed manually. Then, the process continued through the screening of titles and abstracts which was followed by the evaluation of the full text of those works not excluded on the basis of the latter.

**Figure 1:**
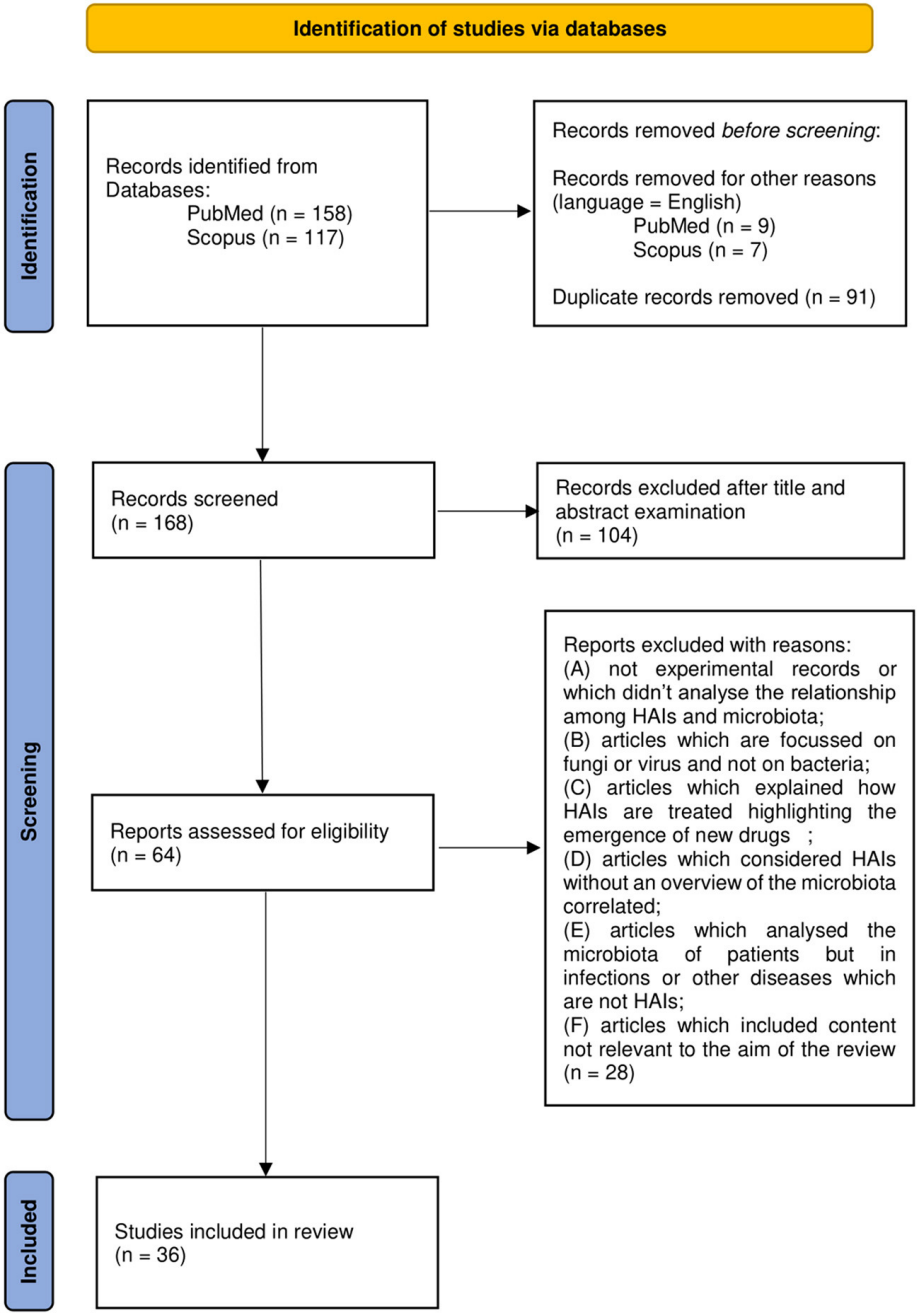
Preferred Reporting Items for Systemic Reviews and Meta-Analyses (PRISMA) 2020 flow diagram.

A total of 165 works were thus examined on the basis of title and abstract. A total of 62 articles were further evaluated by full-text examination to exclude irrelevant content based on the previous criteria (A-F). After a full-text reading of the selected papers, 36 were considered eligible and included in the review. Results management was performed with the use of Microsoft Office software such as Excel and Word. Zotero software was used to edit and organize the bibliography. The PRISMA flow chart in Figure 1 summarizes the workflow of the screening and selection process described above.

## Results

**Figure 2:**
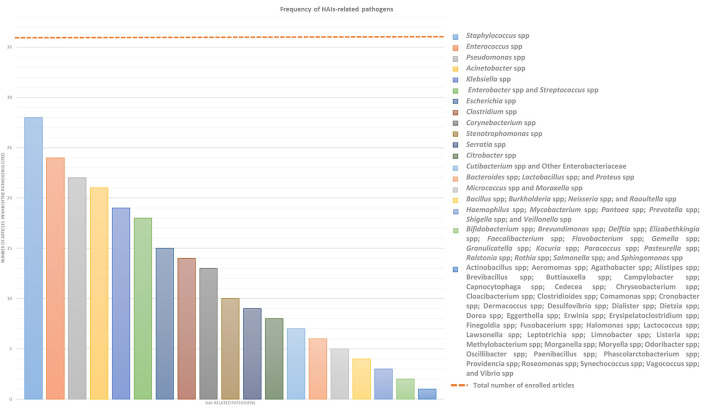
Frequency of HAIs-related pathogens. In the graph are represented the number of article out of the 36 enrolled which treated each specific pathogen to better highlight which of them result most prevalent. ESKAPE bacteria, namely *Enterococcus* spp, *Staphylococcus* spp, *Klebsiella* spp, *Acinetobacter* spp, *Pseudomonas* spp, and Enterobacteriaceae such as *Enterobacter* spp and *Escherichia* spp, in addition to *Streptococcus* spp and *Clostridium* spp resulted the most infective pathogens which should be controlled to prevent HAIs.

This systemic review analyses in detail different studies, which deal with the association between microbiota and HAIs focusing on different types of microbiota, such as that of HCWs, patients, or HE, in order to define alarming factors for HAIs spreading and to identify strategies for HAI control or prevention. Over the years, new technologies such as next-generation sequencing (NGS) technologies have emerged, allowing a deeper and more precise understanding of microbiome in different contexts, providing specific knowledge toward new guidelines for combating HAIs and thus promoting and improving citizens' health. In this sense, a multifaceted approach is required and the analyses of the many factors, which can influence HAIs onset, could be a good starting point. In this review, we will focus mainly on the localization, transmission, and prevention of “ESKAPE” bacteria (*Enterococcus* spp, *Staphylococcus aureus, Klebsiella* spp, *Acinetobacter* spp, *Pseudomonas aeruginosa*, and *Enterobacteriaceae*) and Clostridium difficile, which are the most common pathogens causing HAIs, despite sometimes we will present a wider microbiota landscape (Figure 2). In order to provide the reader with a better understanding, we grouped the selected studies on the basis of the relationship of HAIs with seven categories: (a) HCWs microbiota and HAIs; (b) patients microbiota and HAIs; (c) HE microbiota and HAIs; (d) medical equipment microbiota and HAIs; (e) environmental factors, ecosystem, and HAIs; (f) study of transmission/cleaning and HAIs; and (g) resistome and HAIs. A summary of the results of the studies analyzed in this review is shown in Supplementary Table 1.

### Healthcare workers' microbiota and HAIs

**Figure 3:**
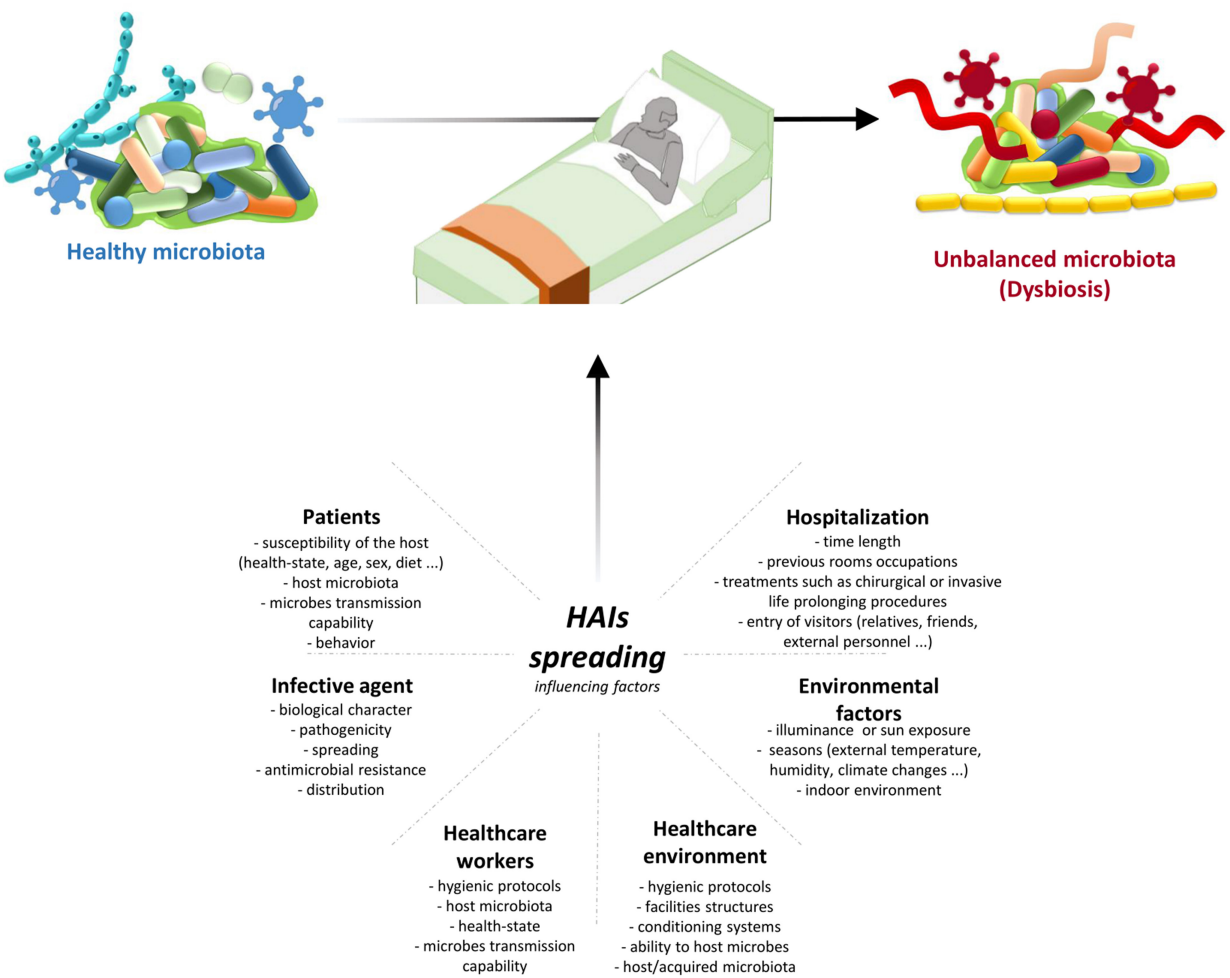
HAIs spreading influencing factors. HAIs are mainly due to the microbiota transition from an healthy state to dysbiosis. This alterations are caused by different factors: hospitalization conditions and reason for admission; both external and internal environmental factors; HE hygienic condition and HE ability to host/acquire microbes; ability to transmit microbes by the HCWs, their healthy condition, and their propensity to follow hygienic procedures; infective agent itself; and patients healthy state and their behaviour. Therefore it is important a multifaceted approach to manage all these factors and thus HAIs spreading. HAIs, healthcare-associated infections; HCWs, healthcare-workers; HE, healthcare-environment.

Transmission of infection during healthcare assistance requires three elements: the source of infecting microorganisms, a susceptible host, and a means of transmission from the microorganism to the host. Infection can be endogenous when the source is represented by pathogens present within the body, but more frequently exogenous. In this case, the infection is transmitted from the outside through medical equipment or devices, the environment, healthcare personnel, or contaminated drugs (Figure 3).

There are many shreds of evidence that HCWs are one of the main risk factors involved in the large-scale dissemination of HAI-related bacteria; therefore, several studies have analyzed their implications in this context. Sereira et al. carried out the “Healthcare-associated Infections Microbiome Project,” a surveillance program of 6-month targeting, among others, HCWs, collecting 216 samples from their hands, mobile phones, and protective clothing. Through their culture-dependent and culture-independent analyses, despite the high abundance of total bacteria in the protective clothing, they observed a similar distribution of the ESKAPE bacteria investigated on the different sources identifying these sites as possible hot spots for HAI-related bacteria transmission (50). As we have previously pointed out, in addition to ESKAPE bacteria, Clostridium difficile is another important HAI-related bacteria. Shoaei et al. specifically have performed phenotypic characterization coupled with molecular typing of Clostridium difficile isolates in burned patients with diarrhea, as well as their environmental context. In particular, from the point of view of HCWs-related Clostridium difficile analyses, they collected 29 swabs from HCWs dominant hands which showed positive results for Clostridium difficile colonization in 8 samples, one of which resulted colonized by a toxigenic Clostridium difficile strain (51). A similar result was highlighted by Segal et al. They identified in an anaesthesiologist the source of contamination of post-operative infections of seven ICU patients, detecting in both patients and the anaesthesiologist the same bacteria. This result was achieved using 16S rRNA amplicon metagenomics sequencing rather than the most common cultural methods. Moreover, they observed also a correlation between the time of surgery and the severity of infection, suggesting that the number of contacts between the patient and any contaminated member of the medical team may increase the possibility for the patient to acquire that particular pathogen (52).

Since HAIs are considered as those infections acquired in any healthcare facilities, which provide healthcare or diagnostic service to individuals, and not only those acquired in hospitals, all the employers of these facilities should be considered, in this context, as HCWs. On this base, Pérez-Fernández et al. performed a descriptive observational study on 19 physiotherapy and rehabilitation centers in order to discover potential microbiological risk factors for HAIs onset. They performed sampling from the hands of physiotherapists without previously informing them to prevent influencing their behavior (hand washing in particular). The majority of the detected microorganisms were gram-positive bacteria, in accordance with the usual microbiota of the human body, suggesting the necessity of reinforcing hand washing or even combining hand washing with the use of gloves to reduce the transmission of these bacteria among different patients (53).

Cruz-López et al., in addition to studying the role of HCWs as a source of external human contamination for HAIs in-patient, have gone over identifying also the huge contribution that can be attributed to the patients' relatives. They performed sampling from 35 nurses and 8 patients' relatives. In particular, stool samples or rectal swabs and swabs of different anatomical sites as well as hands swabs were collected from the HCWs only once during the first seven days of the study, and from the patients' relatives at admission, on day 3 and then every 5 days until hospital discharge. They observed that, among the different microorganisms, coagulase-negative staphylococci represented the most frequent species recovered both in HCWs and in patients' relatives. *Staphylococcus aureus* and Raoultella ornithinolytica were recovered primarily from nurses. However, they identified a wider spectrum of microorganisms that could be present in both HCWs and patients' relatives, identifying them as possible asymptomatic carriers and pathogens reservoirs that can facilitate the dissemination of the pathogens in the hospital setting and that, hence, should undergo strict regulation to prevent HAIs dissemination (54).

Considering this evidence, HCWs and patients' relatives/caregivers must be considered one of the main sources of pathogen spreading in different types of healthcare facilities. More than half of HAIs are preventable, especially those associated with certain behaviors, through the planning of dedicated programs to prevent and control the transmission of infections. However, it is necessary to plan and implement control programs at different levels (national, regional, local), to ensure the implementation of those measures that have proved effective in minimizing the risk of infectious complications. Although HAIs are commonly attributable to patient variables and the quality of care provided, a dedicated organizational setup has been shown to help prevent them. Therefore, ensuring correct hygiene practices of all people involved in the patient's assistance, and the use of sterile gloves and clothing should be a fundamental strategy to ensure a higher safety level for patients, especially for the immunocompromised ones.

### Patients' microbiota and HAIs

Human microbiota has a central role in many biological functions therefore its variations may represent important risk factors for HAIs onset and development. In this paragraph, we are taking into account different studies that considered the patient microbiota in order to define its correlation with HAIs onset from a general point of view or focusing on either specific pathologies or pathogens. McDonald et al. worked to confirm an earlier assumption according to which critical illness would be associated with loss of health-promoting commensal microbes with a simultaneous overgrowth of pathogenic bacteria (dysbiosis), thus increasing susceptibility to HAIs, sepsis, and multi-organ failure. They collected fecal, skin, and oral samples from 115 mixed ICU patients twice: within 48 h of ICU admission and on day 10 or at ICU discharge. First of all, they observed a greater similarity among fecal and oral samples at admission, compared to those obtained at discharge, suggesting that the length of stay in an ICU is connected with endogenous microbial community disruption. Their results confirmed the correlation between critical illness and the rapid establishment of a state of dysbiosis due to the depletion of health-promoting organisms (such as Faecalibacterium which seems to have an anti-inflammatory role), and the overgrowth of known pathogens, such as *Enterobacter* and *Staphylococcus* (55). Sereira et al. in their “Healthcare-associated Infections Microbiome Project,” collected 198 patients' samples through rectal, nasal, and hand swabbing. A high abundance of HAI-related pathogens were detected on all types of samples, however, the highest amount of bacteria and the greatest differences in alpha and beta-diversity was associated with the rectum samples. Moreover, during their studies, they concluded that 50% of the patients did not present any HAIs during hospitalization, 43.9% had an HAI during the hospitalization, and 6.1% was colonized by HAI-related bacteria, highlighting that a longer hospitalization seems to result in an increased HAIs incidence and, thereby, in an increased HAI-related pathogens detection, especially for bacteria like *Klebsiella pneumoniae, Enterobacteriaceae, Staphylococcus* spp, and *Acinetobacter baumannii* (50). Always through samples collected from different patients' body sites, Cruz-López et al. examined the colonization process and the possible ways of transmission of HAI-related pathogens, since patient colonization has been suggested as a risk factor in HAI development. They collected stool samples or rectal swabs and swabs from different anatomical sites in 11 in-hospital patients, at admission, on day 3, and every 5 days until the patient left the unit. Of these patients, 8 developed 1-3 HAIs for a total of 12 diagnosed HAIs. Since the main causative agents were identified in *Acinetobacter baumanii* and *Klebsiella pneumoniae*, the majority of HAIs (50%) were ventilator-associated pneumonia (VAP). Moreover, this study confirms the previous one, according to which the risk for HAIs-related pathogens acquisition increases with the time of hospitalization since on the 1st day of hospitalization only 50% of causative agents were recovered from patients' swabs (54).

Zakharkina et al. performed a study focused on the dynamics of the respiratory microbiome during mechanical ventilation in the ICU and its association with VAP. They collected a total of 111 samples of tracheal aspirates from 35 patients which have been divided into 4 groups: 11 patients with VAP (group 1), 9 patients without VAP but with colonized airways (group 2), 9 patients without VAP and without colonized airways (group 3), and 6 patients who developed pneumonia within 48 h after intubation (group 4). From a microbiological point of view, pathogens like *Acinetobacter, Pseudomonas*, and *Staphylococcus* were identified in patients with confirmed VAP and the duration of mechanical ventilation resulted to be associated with a decrease in microbial diversity in 83% of patients. More in detail, differences in alpha diversity were detected between group 1 and group 3 but not between groups 1 and 2. However, in group 1 patients showed a more profound dysbiosis than in group 2. Moreover, despite 27 patients receiving treatment with antibiotics at some point during their hospitalization, in this case, an association between antibiotic therapy and microbiological variations was not observed (56). Another study addressing microbial variation in mechanical ventilated ICU patients is that of Lamarche et al.; they conducted an observational study collecting samples from 34 mechanically ventilated ICU patients and from 25 healthy adults. In particular, for critically ill patients, they carried out the sampling during the 1st week of hospitalization, collecting 29 endotracheal aspirates, 26 gastric aspirates, and 10 feces specimens; whereas, for healthy adults they collected 7 bronchoalveolar lavages samples, 7 nasopharyngeal swabs, 7 oropharyngeal swabs, and 21 feces specimens. As described above, microbial dysbiosis occurred in critically ill patients, moreover, in this case, less pronounced differences in biogeographical composition, resulting in a lack of specificity between anatomical sites, were observed. Comparing ICU patients and healthy adults' operational taxonomic units (OTUs), significant differences in their abundance were shown with a strong decrease in the amount of the “health-promoting” microorganisms, in particular *Faecalibacterium* and *Neisseria*. Also, this study reiterated that in ICU patients, besides the depletion of different OTUs, there is an increase of one or few pathogenic OTUs (such as *Enterococcus, Pseudomonas*, and *Staphylococcus genera*), suggesting the microbial collapse in ICU patients toward a few dominant taxa which are usually isolated in HAIs and whose number is proportional to illness severity and mortality (22). More recently, Lu et al. focused their attention on patients with severe pneumonia, analyzing their skin microbiota composition and diversity in comparison with that of a healthy control group. They enrolled 30 mechanical ventilated ICU patients and 30 healthy staff members and collected from both groups skin surface samples and then, only from the patients, blood, endotracheal aspirates, and bronchoalveolar lavage fluid samples. From skin surface samples 14292 OTUs were identified, allowing identification of 590 genera. *Staphylococcus, Acinetobacter, Corynebacterium, Stenotrophomonas, Enterococcus, Brevibacillus*, and *Halomonas* were most abundant in the patient group, while in the healthy control group *Bacteroides, Phenylobacterium, Prevotella*, and *Streptococcus* were prevailing. However, as previously observed, patients showed also a decrease in diversity both within each sample (alpha diversity) and between samples (beta diversity). Finally, they showed an interaction between skin bacteria and respiratory microorganisms characterizing also in the other samples the same HAI-related pathogens, thereby suggesting the importance of skin microbiota along with pulmonary and gut microbiotas in the pathogenesis of severe pneumonia in ICU patients (57).

Another observational study is that performed by Mu et al. on three groups of patients (34 septic patients, 33 non-septic ICU patients, and 10 healthy adults) and 312 fecal samples. Despite the focus of their study on the gut microbiota, also in this case a significant decrease in microbiota abundance and diversity, flanked by an increase in AMR pathogens, was highlighted. Moreover, they found an association between opportunistic pathogens intestinal colonization and secondary infection development, observing a secondary infection in 23 septic patients out of 34, 14 of which resulted to be caused by *Klebsiella pneumoniae*, thus suggesting the central role of this pathogen in HAIs. Actually, *Klebsiella pneumoniae* was not the only opportunistic pathogen showing a higher abundance in both septic and non-septic ICU patient than in healthy adults; indeed, also Enterococcus strains were detected. In this study, the amount of other health-promoting bacteria, such as Faecalibacterium, was significantly lower in both septic and non-septic ICU patient than in healthy control (58).

Considering the focus on gut microbiota, in their study Lu et al. explored early intestinal colonization in very low birth weight infants (VLBWI) and how it is influenced by dominant bacteria and other factors. They collected a total of 300 anal swabs from 81 VLBWI at different times after birth until the 21st day of hospitalization. Their results showed that 188 samples out of 300 had dominant bacteria, the top five were both gram-negative bacteria (*Klebsiella pneumoniae, Escherichia coli*, and *Serratia marcescens*) and gram-positive bacteria (*Enterococcus faecalis* and *Enterococcus faecium*). However, the gram-negative bacteria resulted to be the main colonizers in VLBWI with a colonization rate that increased over time. In particular, HAIs due to both *Klebsiella pneumoniae* and *Serratia marcescens* proved to be significantly associated with intestinal colonization rather than those caused by *Escherichia coli* or *Enterobacter cloacae*. Finally, among different non-infectious factors considered in this, including gender, mode of delivery, gestational age, birth weight, feeding mode, and mechanical ventilation, only the latter was shown to be a factor affecting bacterial colonization in VLBWI, which was probably influenced also by the use of antibiotics to treat these patients (59). Also, Maamar et al. faced gut microbiota colonization but they mainly focused on a broad-spectrum cefotazime-resistant (CTX-R) Enterobacteriaceae to determine their prevalence in patients and their colonization rate during hospitalization. They enrolled 63 patients collecting different rectal swabs at admission and on a weekly basis until pathogen positive detection or hospital discharge. Firstly, at admission, 13 samples resulted to be positive for CTX-R *Enterobacteriaceae* indicating a prevalence of 20.63%. The following sampling was realized for only 35 patients, 15 of them acquired the pathogens during hospitalization, resulting in a CTX-R Enterobacteriaceae acquisition rate of 42.85%. In particular, CTX-R *Klebsiella pneumoniae, Escherichia coli*, and *Enterobacter cloacae* were the most frequently detected microorganisms, confirming their pathogenic role. Eventually, also Maamar et al. identified antibiotic treatment as a risk factor for pathogens acquisition (60). Since the gut microbiota of an individual can shape the local environmental surfaces, Freedberg et al. collected 304 samples from ICU patients at the time of ICU admission, 80 of which were defined as eligible to be compared with ICU rooms' microbiota. In addition to these evaluations, Enterococcaceae resulted to be overrepresented in all samples and vancomycin-resistant Enterococcus (VRE) was identified specifically in 28% of the eligible patients (61).

Ke et al. turned their attention to another important HAIs-related pathogen, Clostridium difficile. They recruited 243 participants which have been divided into four groups: 112 patients with Clostridium difficile infection (CDI), 40 asymptomatic carriers, 44 non-CDI patients with diarrhea, and 47 control patients. These authors analyzed not only gut microbial composition but also a broad panel of innate and adaptative immunological markers, suggesting that all these data taken together may allow to better distinguish patients with CDI from other groups of patients. This new association may be a new marker-derived signature to detect CDI and design early and more effective therapeutic interventions. In addition, they compared the overall microbial community structure of the four groups of patients identifying CDI ones as those with the lower alpha diversity and the higher beta diversity. Consistently with previous studies, these findings suggested a depletion of some taxa, and a significantly less stable microbiome profile characterizing CDI patients. Despite this, several driven taxa, like *Klebsiella, Streptococcus, Desulfovibrio*, and *Veillonella*, were identified as the main players in driving changes in microbial correlation networks between CDI patients and other groups and many other genera showed specific variations (62). Considering the role of Clostridium difficile, Shoaei et al. evaluated the dominant bacteria structure in burned patients with and without CDI. They collected fecal samples from 23 CDI patients, 46 burned patients without CDI, and 46 healthy control adults for a total of 189 samples, and 51 skin surface samples from burned patients. Fifty-one fecal samples showed Clostridium difficile positive results with culture methods, of which 23 had toxigenic character whereas, for the second group of samples, 14 showed positivity to Clostridium difficile culture but only two of them produced results showing colonization by toxigenic Clostridium difficile strains. More generally, they demonstrated that the gut microbiota of CDI group was characterized by an overgrowth of facultative anaerobic bacteria such as Enterococcus spp and *Escherichia coli* and a reduction of beneficial bacteria such as Bacteroidetes compared to other groups. Moreover, they identified that the increase in *Akkermansia muciniphila* and the decrease in *Faecalibacterium prausnitzii* may be considered predictive microbial markers for developing nosocomial diarrhea, defining a poor CDI prognosis in burned patients (51).

Ogura et al. enrolled 29 patients to characterize Staphylococcus spp on skin healed from a pressure injury. The patients were divided into two groups since 7 of them suffered from recurrent pressure injury (RPI) within 6 weeks after healing and the other 22 did not. The results showed a significantly higher abundance of *Staphylococcus* spp in RPI-healed sites than in non-RPI-healed sites suggesting its implication in RPI. From a genomic point of view, they demonstrated the dominance of *Staphylococcus caprae* and *Staphylococcus aureus* over *Staphylococcus epidermidis*, whose presence showed extremely low results in all skin sites. Moreover, despite *Staphylococcus aureus* seeming to appear in an earlier RPI onset, it was detected alone in only two of the seven RPI patients in comparison to *Staphylococcus caprae*, which was observed alone four times (63).

Given all these studies and their similarities, it is clear how patient microbial diversity may be considered as a biomarker of prognostic value for HAIs and a starting point to define targeted therapies to correct dysbiosis and health-promoting bacteria depletion, restoring a healthy microbiome and thus improving patient outcome.

### Healthcare environment microbiota and HAIs

Safety and hygiene of HE significantly contribute to the onset of HAIs, indeed different studies identified microbial contamination of the HE as an important source of pathogens transmission resulting in HAIs spreading. The monitoring of HE surfaces may be conducted through either the most common culture-dependent methods or culture-independent ones, which, in general, result to be faster, more effective and sensitive, and able to detect also uncultivable bacteria with the only fault being unable to distinguish viable from dead bacteria, leading to an overestimation of the contamination. Comar et al. performed one study of HE contamination using NGS technologies in comparison with conventional microbiological and molecular PCR methods, in order to define more precisely the environmental microbial composition. They collected HE samples 7 h after cleaning by contact plates for microbiological analyses and sterile swabs both for molecular ones and for NGS analyses. After sampling, 216 contact plates and 108 sterile swabs were harvested to perform microbiological, molecular, and NGS analyses, respectively. In microbiological analyses, Staphylococcus showed a microbial prevalence of 81% of the total collected samples, Enterococcus spp of 13%, Candida spp of 7.9%, Acinetobacter spp of 7.4%, Clostridium difficile of 4.2%, *Pseudomonas aeruginosa* of 0.9% and Klebsiella spp of 0.5%, whereas Aspergillus was never detected. Molecular analyses allowed the identification of the searched pathogens in more samples compared to the previous method. In particular, among the others, Staphylococcus was detected in 99% of the samples, Enterococcus spp in ~80%, Klebsiella pneumonia, and Enterobacter in 78%, often in association with *Escherichia coli* which was identified in 49% of the samples, *Acinetobacter baumannii* in 24%, *Pseudomonas aeruginosa* in 76%, and Clostridium difficile in 19%. NGS analyses allowed obtaining the following level of microbial prevalence for the most frequent pathogens: 94.5% for Cutibacterium spp, 92.6% for Staphylococcus spp, 82.4% for Streptococcus spp, 75% for Corynebacterium spp and Pseudomonas spp, 70.4% for Paracoccus spp, 65.7% for Acinetobacter spp, and 59.3% for Rothia spp. Considering all the results achieved, NGS appeared to be the only technique able to identify both searched and non-searched bacteria, with a high degree of sensitivity compared to the other two techniques, since NGS is a powerful tool for monitoring contaminating bacteria even at low concentrations (13). Another study that exploits NGS potential, rather than cultural methods, is that of Ribeiro et al., where deep-DNA-sequencing analyses were used to explore and compare the bacterial communities structures of different ICUs and neonatal intensive care units (NICUs). For this purpose, 158 samples were collected resulting in identification of 2051 OTUs for NICU and 1586 for ICU, resulting in higher diversity in the microbial composition of NICU compared to ICU, probably due to the higher transit of visitors in the former. At the genus level, sequences of 138 and 160 genera were included for ICU and NICU, respectively, among which 11 specific genera were identified as biomarkers for NICU and 6 for ICU. The HAI-related genera, which were considered biomarkers for the NICU environment by Ribeiro et al., were several facultative or obligate anaerobes, most of which, despite normal hosts of healthy adults, may be pathogenic for neonates. Instead, the main HAI-related pathogens in ICU were Pseudomonas. Collectively, these results enable to differentiate ICU and NICU environments, suggesting the central role of HCW and patients in environmental contamination since the majority of the detected pathogens are common in human microbiota (64).

Moreover, Li et al. conducted monitoring of the microbial community of ICUs. They collected 214 samples from different sites of two ICUs within a 1-year period and, then, they compared the microbial composition detected with public databases to figure out the sources of ICUs contaminations. They identified the main sources of ICUs contamination in building-related bacteria and, to a lesser extent, in human skin-related bacteria. Anyway, in addition to Proteobacteria and Firmicutes, which represent the main phyla of these two ICUs, this study showed a huge HAI-related bacteria composition characterized by Acinetobacter, Staphylococcus, Enterococcus, and Klebsiella strains (15). Again in the ICUs context, Costa et al. analyzed biofilm and ESKAPE bacteria contaminations of high-touched surfaces. Fifty-seven surfaces were selected and the samples were analyzed with four different methodologies (culture, molecular analyses, NGS, and microscopy). ICUs surfaces resulted to be contaminated by many pathogens which were identified mostly through molecular analyses rather than cultural ones, indeed from the culture-negative samples, 76.7% were shown to have live bacteria suggesting the presence of a high number of non-culturable bacteria such as those found in biofilm. Moreover, biofilms were detected in all the analyzed samples through microscopy techniques. Eventually, NGS analyses revealed a large microbial diversity with more than 830 OTUs and 170 genera, among which ESKAPE bacteria were detected in 51.8% of the NGS samples. In particular, among these HAI-related bacteria, Acinetobacter baumanii was detected in six culture-positive and five culture-negative samples, *Staphylococcus aureus* in three culture-positive and one culture-negative, Enterobacter spp in two culture-negative, and *Pseudomonas aeruginosa* in one culture-negative sample (65).

Sereira et al. during their “Healthcare-associated Infections Microbiome Project” targeted also HE in order to identify contamination hotspots, searching for specific bacteria in 666 high-touched surfaces samples. Collectively they showed that the microbial community in HE was mainly composed of *Proteobacteria* and *Firmicutes phyla*, where *Enterobacteriaceae, Pseudomonas, Acinetobacter baumanii*, and *Escherichia coli* belonged to the first phylum and Staphylococcus to the second one. Concerning hotspot sites, HCW resting rooms resulted as the most contaminated, with a high amount of both total bacteria and HAI-related bacteria. Moreover, higher diversity was shown in the unit's bathrooms as well as in bed equipment and equipment shared between hospital units (50).

Differently from Ribeiro et al. who concluded that ICUs and NICUs environment are characterized by different microbial compositions, Sereira et al. affirmed that these differences disappear over time due to the microbial community dynamicity, especially when a larger sampling size is adopted for the analyses. However, in accordance with Ribeiro et al., and Sereira et al. suggested that microbial communities which colonized HE can be influenced by patients, HCW, and the severity of illness of inpatients (50, 64). Another study, which identified in HE microbiota a possible transmission route of HAI-related pathogens, is that of Cruz-López et al. which collected environmental samples from surfaces near the patient's bed at admission, at day 3, and every 5 days until the patient's discharge. Coagulase-negative staphylococci were the most detected species also in environmental samples; however, other HAI-related bacteria were identified, such as Acinetobacter baumanii, *Klebsiella pneumoniae, Enterobacter cloacae*, and Enterococcus spp. HAI causative agents were recovered both before and after infection development suggesting a mutual exchange of bacteria between patients and the environment (54).

Kelly et al. studied how HE contamination may be related to the environmental position of patients and wastewater sites. They considered 51 hospital rooms at the time of patients' admission with an eligible HAI-related pathogen and then they performed a longitudinal sampling at different times in three different sites at variable distances from the patient's bed (near to the patient, intermediate distance, and far from the patient but in the proximity of wastewater sites) resulting in 408 samples. They related the probability of HAI-pathogens detection to the distance from the patient and wastewater site evidencing that the detection of gram-negative HAI-related pathogens (such as *Acinetobacter* spp or *Pseudomonas* spp) increased toward the wastewater site, while the opposite occurred for the detection of gram-positive HAI-related pathogens (such as Clostridium difficile) which increased closer to the patient. The relation between pathogens and the distance from the patient may be helpful to evinced possible hotspots of bacterial contamination (66).

Gudakova et al. analyzed microbial contamination specifically on touch surfaces of waiting rooms in pediatric outpatient facilities, to evaluate any differences between sick-child waiting rooms and well-child waiting rooms and possible hotspot sites of contamination. They collected samples from 3 pediatric offices in one or two sampling days. Taken together, their results revealed no significant differences between the two types of waiting rooms, both characterized by a high variation in microbial burden on samples collected from the same surface type. However, they highlighted that the sites with the highest microbial contamination were seats, children's seats, and children's books. Seats hosted the highest levels of *Staphylococci*, whereas children's books showed the highest level of both *Staphylococci* and gram-negative enteric bacteria. Moreover, they noted that the level of seat contamination was higher in sick-child waiting rooms in contrast to the level of children's books contamination, the results of which were higher in well-child waiting rooms. A probable explanation of these results may be connected with the different behavior of the children in the waiting rooms which is influenced by their health state (67).

A particular consideration is that also ambulances can be categorized within HE. In this context, Sheahan et al. developed a rapid, portable, inexpensive, and easy-to-use approach to metagenomics analyses to characterize ambulances microbiota. Their system allowed them to identify, on the samples collected from different ambulances at different times, six different phyla (*Spirochaetes, Fusobacteria, Bacteroidetes, Actinobacteria, Firmicutes*, and *Proteobacteria*) for a total of 68 genera, some of which contain HAI-related pathogens, such as Clostridium spp or Staphylococcus spp. In addition, other identified genera are: Campylobacter, which is a bacteria responsible for some gastroenteritis; Shigella which is associated with shigellosis disease; and Listeria, which may lead to fatal bacterial illness. Finally, by analyzing different surface samples from three different ambulances they were able to detect probable contamination hotspots, which should require fine monitoring and cleaning procedures. Overall, their approach provided a functional and rapid platform for microbial detection and monitoring in ambulances for specific pathogens, evidencing their higher prevalence on finger monitors which enter in direct contact with patients, followed by door handles which experience direct contact with the HCW, and by soft kits which are in contact with the environment (68).

Other healthcare facilities analyzed in order to discover environmental contaminations were physiotherapy and rehabilitation centers. Pérez-Fernández et al. collected four environmental samples from each of the 19 healthcare facilities under study, in particular, three samples were taken from the treatment table (head, intermediate, and caudal section) whereas the fourth sample was of the ambient air. They observed a high value of coagulase-negative staphylococci and gram-negative non-Enterobacteriaceae bacteria but lower level of *Staphylococcus aureus* on samples collected from tables of treatment without any relevant differences among the different tables' sections. On the contrary, *Staphylococcus epidermidis, Micrococcus* spp, and *Bacillus* spp were the only microorganisms identified in air samples. Their results suggested greater involvement of environmental surfaces rather than the ambient air in pathogen transmission since the former was shown to host opportunistic pathogens (53).

In addition to HCW and patient microbiota analyses, Shoaei et al. performed characterization of environment microbiota in rooms of burned patients after Clostridium difficile diagnosis. Of 21 bed sheets collected samples, three resulted colonized by non-toxigenic Clostridium difficile strains, therefore also in this case a correlation between environmental contamination and HAIs, such as CDI, was highlighted (51). Considering environmental contamination and VRE colonization, Freedberg et al. tried to define if there might be worse ICU rooms. Twenty-four ICU rooms were sampled at five different time points. Pseufomonaceae characterized the microbial community detected on environmental surfaces. Moreover, comparative studies to assess microbial variation across neighboring ICU rooms were performed. The rooms' microbiota slowly diverged from baseline and it appeared similar to that of neighboring rooms; however, the speed of this divergence seemed to be associated with the patients' turnover. Moreover, analyzing VRE-colonization, the authors confirmed environment-patient interactions, indeed when they showed a different VRE status in a time of 3/9 days both become VRE-positive (61).

Taken together all these studies allowed defining the central role of HE microbiota in the transmission of different HAI-related pathogens, most of which are not common environmental bacteria but rather are human-related bacteria released in the environment by the individuals who spend time in that environment.

### Medical equipment microbiota and HAIs

Another important cause of HAIs has been recognized in the contamination of medical devices by pathogenic microorganisms in healthcare settings. Therefore, to reduce the burden of HAIs, accurate studies to characterize the microbiota of medical devices and to identify the main sources that lead to their contamination may be other important interventions to enhance the effectiveness of infection prevention and control practices. Therefore, different scholars performed studies to this end. Among them, Shoaei et al. were the only ones that, analyzing 19 samples from medical devices in hospital rooms of burned patients affected by Clostridium difficile, and did not find any positive contamination from Clostridium difficile (51). Opposite results were obtained by Pérez-Fernández et al. in their observational study on 19 physiotherapy and rehabilitation centers, in which the level of contamination on instruments and equipment used for patient therapies administration was investigated. They detected a greater presence of Enterobacteriaceae as well as *Staphylococcus epidermidis* in different devices. In addition to these bacteria, Pérez-Fernández et al. identified other important pathogens, such as *Staphylococcus aureus*, Acinetobacter spp, and *Escherichia coli* on the sponge electrode. In this study, sponge electrodes represented the instrumental samples with higher and more varied contamination, up to more than 20 different bacterial species, probably as the consequence of inadequate cleaning (53).

In addition to previous studies, Cruz-López et al. examined the colonization process and the possible transmission routes of HAI causative agents through the sampling of medical devices, such as mechanical ventilation tubes, central venous catheters, and urinary catheters. They collected samples from medical devices near to patients at different times, on day 1 of admission, day 3, and every 5 days until the patient's discharge. The same HAI pathogens identified in patients were also identified on medical devices and in particular mechanical ventilation tubes were the most colonized medical devices in those patients that developed VAP between day 1 and day 3 (54).

In their study, Mahjoub et al. worked analyzing the instrumentations of ophthalmology clinics to identify potential sources of pathogenic spread. The collection of the 33 samples was performed at 6 am before any patients or staff members entered the clinics and after the cleaning of the night before. More than half samples yielded bacterial growth, without significant differences among the clinics. Different pathogens were detected, first of all, *Staphylococcus epidermidis*, associated with post-intraocular surgical infection; followed by *Staphylococcus capitis*, implicated in surface infection such as purulent conjunctivitis; Micrococcus luteus, able to form biofilms implicated in prosthetic valve endocarditis; Corynebacterium species, causing granulomatous mastitis, and, moreover, Cutibacterium acnes, which is well-established as a cause of post-operative chronic endophthalmitis. Therefore, these findings defined medical devices as possible vectors for HAIs spreading indicating a need for increased disinfection of these instrumentations (69). Eventually, Swanson et al. performed a little different study with the aim to identify the main sources that lead to medical device contamination in addition to the characterization of the contamination itself. They used SourceTracker, a DNA sequence-based analytical tool, to identify the sources of contamination of nebulizer devices using the samples' microbiome as a biomarker. They performed source identification to look for four potential sources of microbial contamination: human gut microbiota, human oral microbiota, human skin microbiota, and hospital indoor environment microbiota. The latter was identified as the primary source of microbial contamination in nebulizers, contributing to ~41.3% of microbiomes with a variation ranging from 20.2% to 64.8%. On the contrary, the microbiota from human sources accounted only for ~10% of nebulizer microbiomes, with a higher prevalence referable to the human skin microbiota, followed, by human oral microbiota and human gut microbiota. However, their classification lacking some microbial sources since ~50% of the compounds were not classified as belonging to one of the 4 groups (70).

Based on all these studies, medical devices can represent a possible way for pathogen transmission and HAIs to spread mainly due to wrong cleaning procedures, therefore infection control practices should be developed and implemented to mitigate microbial contamination of medical devices whatever the source.

### Environmental factors, ecosystem, and HAIs

At present, different factors can influence HAIs. From an environmental point of view, it is possible to go beyond the HE, analyzing different environmental factors which characterize our ecosystems, such as humidity, temperature, illuminance, season, and climate changes. The following studies focused on one or more of these factors, contributing to identifying their roles in spreading HAIs, in order to develop control procedures to manage and limit the risk to human health. One of the first studies was that of Ramos et al., which characterized the indoor environmental variations in which microbial samples were taken for the “Hospital Microbiome Project.” This project was designed to investigate microbial community and environmental factors inside 10 patients' rooms and two nearby nurse stations for a period of 1 year in a newly established hospital. Both surface-bound and airborne microbes were influenced by different environmental factors (temperature, relative humidity, and humidity ratio) in their growth or survival responses. Moreover, these Authors observed variation due to light conditions, in particular, because a high degree of sunlight illumination may inhibit bacterial growth or have bactericidal powers. They also observed further correlations with the entrance of the new occupancy and activity explained in the rooms, which may represent the main cause of contamination from human-related microbial communities (71). As well as the previous study, also Freedberg et al. highlighted, in their conclusions, that the microbial community in a healthcare setting may be altered by multiple environmental factors, such as seasonal shifts, solar exposure, and temperature (61). Another study focused on temporal variation is that of Schwab et al. which evaluated the implications of seasonal variations specifically on nosocomial bloodstream infections (BSIs). They performed a retrospective cohort study based on 2 databases (one for HAIs monitoring and one with aggregated monthly climate data) collecting information on about 1196 ICUs located in 779 hospitals and in 728 different postal codes in Germany. Collectively they analyzed more than 6.5 million ICU patients and more than 19000 BSIs in a 15-year period. Through their studies, Schwab et al. were able to determine that the incidence of BSIs was correlated with temperature and vapor pressure, and inversely with relative humidity. Related to temperature the incidence of BSI was 17% higher in months with temperature ≥20°C compared to months with temperature < 5°C. In particular, a strong correlation was observed when the mean monthly temperature of the month prior to the BSI occurrence was considered rather than the temperature of the month of occurrence. More in detail, the gram-negative bacteria were those with the most prominent effect despite the majority of bacteria increased with rising temperatures. *Enterococci* showed no seasonality while Staphylococcus pneumoniae reached a peak in wintertime. These conclusions agreed with previous studies which claimed that gram-negative BSI was most frequently in warmer months; gram-positive BSIs were inconsistent except for *Staphylococcus pneumoniae* BSIs which resulted most frequently in months with the lowest temperatures (72). A similar retrospective observational study, but on another type of HAIs, was that performed by Aghdassi et al., which included more than 2 million procedures resulting in ~32,000 surgical site infections (SSIs) from 1455 surgical departments. They matched the date of the procedures with the meteorological conditions for the month in which the procedure was performed. In accordance with the previous study also SSIs resulted most frequently in the months with temperature ≥20°C rather than in those with temperature < 5°C with a higher correlation for those SSIs due to gram-negative bacteria. This was particularly prominent for Acinetobacter spp and Enterobacter spp for which was shown that a rise of only 1°C led to an increase in SSIs incidence of 6% and 4%, respectively. Among gram-positive bacteria, *Staphylococcus aureus* showed a stronger association with warmer temperatures. However, despite one could think that a stronger correlation should be related to human skin bacteria (such as the latter), a higher correlation between temperature and pathogens was observed for those microorganisms abundant in the human gut (such as *Acinetobacter* spp, *Enterobacter* spp, *Pseudomonas aeruginosa, Enterococcus* spp, and *Escherichia coli*) thereby emphasizing the importance of human gut microbiome also in HAIs pathogenesis (73). More recently, Li et al. demonstrated a significant difference in the microbial composition in healthcare settings on a seasonal time scale. However, despite some HAI-related bacteria such as *Acinetobacter, Pseudomonas, Enterococcus, Staphylococcus*, and *Escherichia* existing throughout the year, they observed their increase in some periods of the year, for instance, Acinetobacter was highly abundant in June and December, whereas Pseudomonas in March, April, and May (15).

In addition, Sereira et al. showed also a specific correlation between months and bacteria amount. In particular, they noted a higher median amount of HAI-related pathogens in May and September, in some sampling sites, due to an increase in *Escherichia coli* and an outbreak of Acinetobacter baumannii, respectively (50). Wu et al. characterized bacterial dynamics among the seasons collecting 10 hospital ambient air particulate matter (PM2.5) samples in summer and 9 in winter. Differently from Proteobacteria, which remain consistent through the entire sampling period, they showed a decrease of 12% in Actinobacteria phylum from summer to winter, and an increase of Firmicutes phyla which passed from 22 to 40%. More generally, the microbiota results detected were less diverse in winter by one order of magnitude overall (74).

All these studies, taken together, indicate that meteorological factors impact microbiological composition and thus may influence the occurrence of different HAIs. Therefore, based on these considerations should be developed proper protocols to control HAI-related pathogens adjusted by months.

### Studies of transmission/cleaning and HAIs

Scientific evidence shows that the application of appropriate cleaning procedures together with campaigns to raise awareness for hand hygiene may lead to reduce microbial contamination and HAIs spreading. Indeed, different scholars, such as Pérez-Fernández et al., concluded that the accumulation and proliferation of HAI-related pathogens might be due to the absence of adequate cleaning and maintenance procedures. In particular, they stated the importance of disinfection not only for HCW, but also for the entire HE including all instruments, equipment, and anything else that has come into contact with the patient (53).

We started considering three articles that tested bacterial transmission alone or in association with cleaning procedures. Del Campo et al. enrolled 30 healthy volunteers (20 women and 10 men) to perform a four-sequential steps protocol of finger-to-finger contact in the same person artificially infected with a precise bacterial inoculum. After the experimental procedure, the volunteers were grouped into three categories, based on their propensity to finger-to-finger bacteria transmission: women were classified in the medium category, whereas the men were divided into the poor or high categories. Analyzing specifically five different HAIs-related bacteria, they defined that gram-positive bacteria such as *Enterococcus faecium* and *Staphylococcus aureus* were characterized by a higher transmission efficiency in comparison to gram-negative bacteria. In particular, despite *Escherichia coli* results showing it to be a ubiquitous bacteria, it was characterized by a low transmission efficiency. Moreover, they performed a second experiment to test the inter-individual transmission chain exploring the finger-to-finger bacterial transmission with all possible combinations of individuals belonging to the three classes; from this test, they detected a reproducible transmission pattern whose efficiency was strictly dependent on the position of the poor transmitter, who cut off the transmission chain (75).

Weber et al. simulated the transmission of ESKAPE pathogens and Clostridium difficile under varying contact scenarios. They performed experiments for both direct (skin-to-skin) and indirect (skin-to-formite-to-skin) transmission by inoculating synthetic skin surrogates with a background skin microbiota or with both background skin microbiota and pathogens, simulating the transmission both before and after cleaning procedures. They observed a higher direct transfer, with smaller differences at low inoculum, compared to those at higher inoculum, for Acinetobacter baumannii, Enterobacter aerogenes, Klebsiella pneumonia, Pseudomonas aeruginosa, *Staphylococcus aureus*, and *Enterococcus faecium*, whereas no significant differences for Clostridium difficile and *Enterobacter cloacae* were observed. In comparison to direct transfer, indirect transfer gave significantly lower transmission rates, except for *Staphylococcus aureus*. Moreover, when decontamination was also investigated, greater differences were observable in the indirect transmission rather than in direct transmission, with a reduction in the transfer of some HAI-related bacteria (76).

Herruzo-Cabrera et al. compared the effect of classic handwashing on native and acquired microbiota with different alcohol solutions. They performed an “*in vitro*” test to evaluate the microbicide effect of the disinfectants on pig skin carrier models, an “*in vivo*” test on healthy volunteers comparing the hands microbiota collected before or after the cleaning, and a similar “field assay” but on HCW in a hospital ICU. Overall, they observed a high reduction of acquired and native hand microbiota (in particular for *Staphylococcus aureus* and gram-negative bacteria) for hands treated with different alcohol solutions. Conversely, only small variations were observed both in native and acquired microbiota after the common handwashing procedures. Therefore, the use of alcohol solution with some detergents or emollients can be more efficient to reduce HAIs, controlling the bacterial-hands spreading (19). Similarly, Wiemken and Ericsson studied the impact of one chlorhexidine gluconate (CHG) application on skin microbiota. They enrolled five healthy adults to analyze their skin microbiota before and after multiple time points after the CHG bathing. No significant evidence were detected in either the short or long term after single CHG use, probably due to the wide broad-spectrum activity which led to an equal reduction of different taxa without eliminating any of them. This suggests the long-period stability of skin microbiota even after a single application of CHG (77).

Differently, Ribeiro et al. analyzed the limits of cleaning procedures in ICU collecting environmental samples both before and after cleaning. Some bacteria decreased after cleaning: indeed, whereas 117 genera were detected before cleaning, only 94 were detected after it. Moreover, despite an overall decrease in diversity associated with a decrease of some genera (HAI-related genera included), some bacteria (such as *Bacillus, Staphylococcus*, and *Acinetobacter*) resulted to be still relatively abundant and sometimes increased. These results highlighted the limitations of the cleaning procedures, since the increase of specific genera, some of which are HAI-related (64). Also, Perry-Dow et al. focused their efforts to characterize the microbial communities of disinfected environmental surfaces. Using NGS, they analyzed two different composite samples collected from 94 rooms post-routine or terminal cleaning with bleach, quaternary ammonium compound (QAC), or a combination of the two. Among the most abundant OTUs detected, gram-negative bacteria (including enteric bacteria such as Enterobacteriaceae) resulted most abundant in QAC-cleaned rooms, whereas gram-positive bacteria (including skin microbiota bacteria such as Corynebacteriaceae) in bleach-cleaned rooms. Instead, a relative lower abundance in Enterobacteriaceae and Moraxellaceae OTUs was associated with rooms cleaned with both QAC and bleach. All these data, taken together, suggested the importance of disinfection to reduce HAIs-related pathogens' surface persistence due to the different impacts of each disinfectant on the different bacteria (78). Additionally, Sheahan et al. concluded that, after cleaning, some bacteria may persist in the environment, further suggesting the different effects of disinfection based on bacteria sensitivity. Moreover, they highlighted that, since it is not possible to ensure a sterile workplace evermore, careful monitoring may aid to develop proper cleaning procedures based on the type of contamination (68).

Differently, Valeriani et al. performed their study on dental mirrors through two different experiments: in the first one, dental mirrors were contaminated by two different salivary solutions, and then six different sanitation procedures were applied; in the second dental mirrors used in care settings were sampled at different steps of the sanitation procedures. Overall, only the dental mirrors which underwent a complete sanitation procedure resulted negative for bacteria, whereas those contaminated or partially sanitized resulted to be positive. This suggested that the analyses of residual traces of a biological fluid microflora DNA might be an important monitoring system of correct sanitation. However, a negative result was mainly associated with culture analyses rather than molecular ones, indeed some negative culture-based microbiological samples resulted in positive to real-time PCR (14).

As we have seen, different studies demonstrated that HAI-related bacteria may persist on environmental surfaces also after cleaning procedures. Caselli et al. proposed new cleaning methods based on addition of healthy-probiotics to hospital surfaces to fight against pathogenic species. Cleaning was performed with Probiotic Cleaning Hygiene System (PCHS) by using detergents containing spores of *Bacillus subtilis, Bacillus pumilus*, and *Bacillus megaterium*. Surface samples were collected before the treatment and on a monthly base for the following 4 months. They monitored different HAI-related pathogens (among the others, *Staphylococcus* spp, *Acinetobacter, Pseudomonas* spp, and *Clostridium* spp) for which a strong decrease after the PCHS treatment was observed. The only exception was the Enterobacteriaceae group, which continued to be scarcely represented over time. Their decrease was evident 1 month after the PCHS treatment and was maintained constant. Probably the microbial decrease may be attributed to PCHS-Bacillus which, reaching 70% of the total microbiota already in 1 month, replaced most of the microbial species originally present on the surfaces, including the pathogenic ones (79). Similarly, Soffritti et al. applied the PCHS to confirm its previously shown ability to decrease the level of pathogens, also in pediatric hospital units. In their experiment, they replaced the conventional sanitation procedure with PCHS treatment for 2 months collecting and characterizing the microbiota with both culture and molecular tests, before and after the PCHS treatment. As in the previous study, they highlighted a microbial contamination reduction, with a simultaneous increase of Bacillus species, which replace the pathogen ones inhibiting their growth. In particular, before PCHS introduction, a high burden of HAI-related pathogens, such as Staphylococcus spp, Pseudomonas spp, Clostridium difficile, and Enterococcus spp and a very low amount of Bacillus spp, were detected. However, after 2 weeks significant changes were observed: Baciullus spp increased, representing 69.9% of all the microbial community, whereas other bacteria diminished. They also highlighted a microbial contamination reduction, with a simultaneous increase of Bacillus species which replace the pathogen ones inhibiting their growth (80). Both these last studies taken together suggest the greater potential of Bacillus-based cleaning procedures compared to the most common procedure used for sanitation, which are usually based on surface sterilization leading to increase resistance and pathogenic bacteria (79, 80).

**Figure 4:**
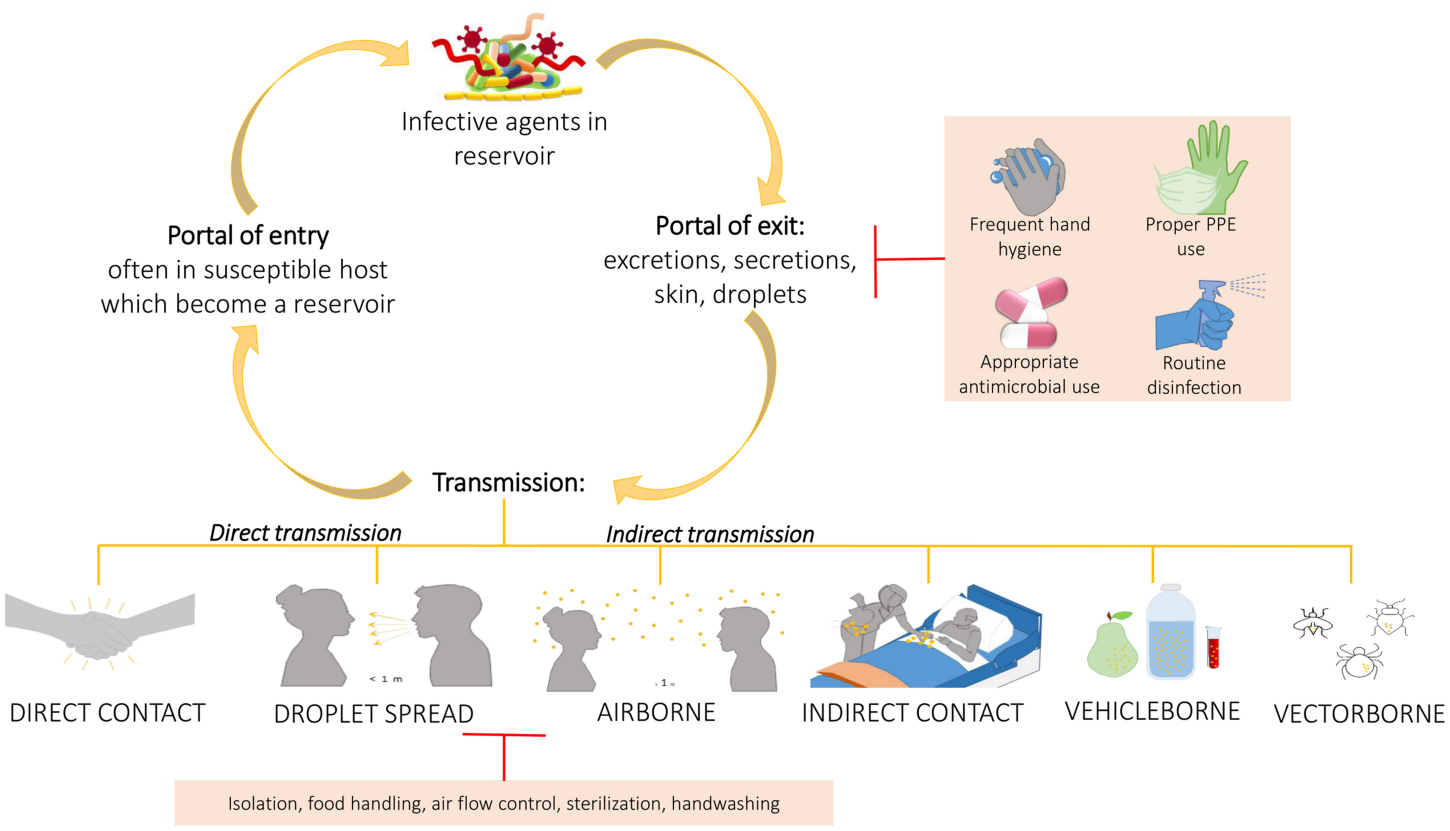
Chain of infection and some preventing strategies. A pathogen may be transmitted from a reservoir to susceptible individual through different ways. Understand the chain of infection is fundamental to HAIs prevention and control. In direct transmission, the pathogens is transferred from a reservoir to an host by direct contact such as skin-to-skin contact, or droplet spread where aerosols produced by coughing, sneezing, or even talking lead to pathogens spreading. Conversely, indirect transmission is associated to an intermediary which may be both animate (vectors) or inanimate (vehicles). Belong to this class the airborne transmission where the pathogens are included into the droplet nuclei suspended in air, this is similar to the previous but the spreading may occurs also some distance away from the source resident due to conditioner systems or air currents. In the transmission through indirect contact, different objects, such as medical devices, contaminated gloves, object in the patients' room or environment and/or medical equipment, may represent the source of contamination as well as inanimate vehicles such as food, water or other biological samples. Sometimes the vehicles are passively, other times they may aid the pathogens providing environment for growth. Similarly occurs for vectors which in general are animals such as mosquitoes, fleas, and ticks. The chain of infection may be interrupted at the “portal of exit” applying hygienic procedure such as the frequent hand hygiene, using proper personal protective equipment, performing proper routine disinfection, or using appropriately the antimicrobial to avoid strengthen antimicrobial resistance of pathogens. Sometime it is possible, instead, to break the transmission limiting the transmission itself through isolation of the infected individual, through a correct food handling, or through the application of sterilization and hygienic procedure.

Based on all these studies, it is important to develop more and more robust cleaning procedures to help management of HAIs, since the use of common cleaning procedures, such as those based on chemicals compounds, may not only let some bacteria on the surfaces (HAI-related ones included) but do not prevent recontamination phenomena, leading to the selection of resistant strains. This was sustained also by Costa et al. who showed also the incorporation of these resistant bacteria in biofilms whose persistence seemed to be not so influenced by the cleaning procedure (65). Given the improper cleaning procedure, the prior presence of patient-carriers of HAI-related bacteria increases the possibility to acquire those same bacteria by the patients who will be subsequently admitted in the same rooms. This further suggests the need to improve environmental hygiene by using a wider spectrum of cleaning or adding beneficial microbes that compete with the pathogenic ones, replacing them (Figure 4). In addition, performing intermedia cleaning during patients' hospitalization rather than only terminal cleanings as proposed by Freedberg et al. (61), or implementing cleaning procedures for those elements for which are not envisaged or, moreover, using instrumentation/structures/elements done with materials easier to clean as sustained by Gudokova et al. (67) may be further aids to HAIs-bacteria management.

### Resistome and HAIs

Cleaning procedures have a starring role in pathogens transmission because either the wrong procedures or the use of chemical disinfectants may cause problems in controlling pathogen contamination, not only in terms of recontamination but also in terms of resistant strains selection. Therefore, identifying the main resistome profiles may give the bases for developing new strategies against resistant pathogens. In Maamar et al., 35 CTX-R Enterobacteriaceae strains were isolated in 28 patients. These isolates were screened for extended-spectrum beta-lactamases (ESBL)-phenotype by double-disk synergy test (DDST) with different antibiotics (ceftazidime, cefatoxime, and amoxicillin-clavulanic acid) disks. Only one isolate was classified as an AmpC producer due to its negative ESBL phenotype with resistance to amoxicillin-clavulanic acid and to cefoxitin, the 34 remaining were classified as ESBL producers. Among them, three resulted with both phenotypes AmpC and ESBL. More in detail, a careful antimicrobial susceptibility test was performed, and all the isolates results were resistant to chloramphenicol, 32 resistant to nalidixin acid, 31 to ciprofloxacin, 29 to three different types of antibiotics (tobramycin, trimethoprim-sulfamethoxazole, and tetracyclin), 27 to gentamicin, 24 to cefoxitin, 22 to ertapenem, and 19 to imipenem. The different strains were analyzed and different resistance genes resulted in transferable by conjugation or co-transferred together. Therefore, most of the CTX-R Enterobacteriaceae strains resulted to be multidrug-resistant (MDR) bacteria, characterized by multiple resistance determinants which cause serious complications for patients limiting the therapeutic options for HAI treatment (60). Cruz-López et al. tested the antimicrobial susceptibility of the different 12 HAI causative agents identified during their study. Eleven of them were MDR bacteria and Acinetobacter baumanii showed resistance to ciprofloxacin, ceftazidime, meropenem, tetracycline, trimethoprimsulfamethoxazole whereas two of three studied *Klebsiella pneumoniae* isolates were carbapenemase producers but all results denoted ESBL producers as well as Raoultella ornithinolytica. Moreover, whereas coagulase-negative staphylococci isolates were resistant to oxacillin and *Staphylococcus hominis* to linezolid, *Enterobacter cloacae* was the only susceptible to all tested antimicrobial agents (54).

Comar et al. analyzed the resistome of the contaminating population through PCR to provide means for the control of HAI transmission. The detected and quantified 84 AMR genes such as those for methicillin, macrolides, beta-lactams (including carbapenems and erythromycin) highlighted the presence of strains resistant to these classes of antibiotics in the analyzed samples. These results allowed them to design specific interventions to fight AMR spreading, especially based on the amount and type of contamination (13). AMR spreading was investigated by Sereira et al. who defined AMR as widely distributed in patients, HE and HCW identified different hotspots of contamination (such as bed equipment, bed bathrooms, and HCW resting areas). In these sites, *Acinetobacter baumanii, Klebsiella pneumoniae, Enterobacter cloacae*, and *Escherichia coli* were identified as the most frequent AMR bacteria. AMR profiles supported these results with the detection of beta-lactamase genes, MDR, extended-spectrum cephalosporin resistance, and carbopenem resistance (50).

Caselli et al. did not limit their study to the resistome profile of a specific microbial community but also studied also its remodeling over time, analyzing the total microbial DNA extracted from the samples detecting and quantifying simultaneously 84 different AMR genes. In the beginning, several resistance genes (against beta-lactams, macrolides, quinolones, and methicillin) were detected in the samples. One month after the PCHS application, these genes decreased. These data were further confirmed through subsequent samplings, with the sole exception of the macrolides resistance gene which resulted in increases every time. This is easily explainable because this resistance gene has been constitutively identified in PCHS Bacillus species, which increase over time after PCHS application and do not acquire other new resistance over time (79). As stated above, a similar study was performed by Soffritti et al. They analyzed the entire resistome both before and after PCHS application in a children's hospital looking for 84 AMR genes. They provided evidence of resistance against macrolides, erythromycin, streptomycin/spectinomycin, erythromycin, beta-lactams, tetracyclin, fluoroquinolones, and methicillin before PCHS application decreasing by an up to 2 logs after the probiotic-based sanitation (80). Thus, confirming what was previously highlighted by Caselli et al. (79).

Whereas, the two previous studies were aimed at characterizing the resistome of the entire microbial community or, at most, those of Bacillus species, Shoaei et al. focused their studies on the AMR of Clostridium difficile isolates with different antibiotics showing their susceptibility for vancomycin and metronidazole and their resistance for moxifloxacin and clindamycin (51).

Wu et al. shifted their attention to the inhalable antibiotic resistome, spreading in healthcare settings through airborne fine PM2.5. In this type of sample, compared to urban ambient air PM2.5, the number of antibiotic resistance genes (ARGs) were nearly doubled, with the prevalence of potential pathogens bacteria of human origin such as Staphylococcus spp and Corynebacterium spp, most of which are MDR bacteria. Among the others, the major resistome components encoded by ARGs were those to aminoglycoside, macrolide-lincosamide-streptogramin, tetracycline, and beta-lactam, whereas the minor ones were bacitracin, rifamycin, sulphonamide (glyco)peptide, and fluoroquinolone. However, collectively, the hospital-specific resistome was significantly associated with the dynamic variation of the bacterial community structure, and the presence of ARG-carrying bacteria in hospital airborne PM2.5 resulted influenced by the HAI spreading (74).

Overall, based on these studies, it is evident how MDR or even pan-drug resistant bacteria cause an increasing number of HAIs, thereby AMRs are becoming a worldwide-relevant problem. Therefore, the understanding of bacteria resistome is of fundamental importance to define new therapeutic strategies to fight against HAIs.

## Discussion

In recent years, a strong focus has been placed on the prevention and control of these infections due to a constantly growing epidemiological trend with strong repercussions on the health of the patients, as well as on the psychological and financial aspects which translate into a prolongation of the length of hospitalization, long-term disability, increased mortality, and spread of antibiotic resistance. The spread of nosocomial infections and multi-resistant microorganisms represent a global health and development threat, especially in the context of HAIs. This is particularly dangerous in healthcare settings due to the diffused and wrong utilization of antimicrobials, which exercise a huge selective pressure on microbes making them stronger and thus therapies ineffective against infections (11, 13). Moreover, for a long time, cleaning was considered mostly an aesthetic requirement rather than an important safety protocol for managing HAIs, however, potential pathogens are not necessarily associated with evident dirt. Indeed, microorganisms survive for a long time on surfaces and specific cleaning procedures can lead to an increase in the number of pathogenic strains over the benign ones, rather than complete surface sterilization. Therefore, sometimes a treatment that increases the number of healthy microorganisms rather than an incomplete surface cleaning, which increases the number of resistant microorganisms, could be a better solution (11, 20). The increase of AMR microorganisms did lead to a change in the causative pathogens responsible for HAIs. Until the beginning of the 80's years, HAIs were mainly due to gram-negative bacteria (such as *Escherichia coli* and Klebsiella pneumonia). One of the challenges still open for the protection of public health is to investigate and identify the variables that influence the risk of HAIs and implement corrective actions to improve the care process, thus reducing the percentage of infected patients (81). Great progress has been made in recent years in the knowledge of the composition (microbiota) and gene expression (microbiome) of the microbial component associated with various body parts (intestinal, respiratory, skin, vaginal, oral, etc.). The advent and continuous development of “meta-omics” and computational technologies is providing revolutionary tools for the study of the microbiota and microbiome, highlighting many aspects inherent to its modulation and the multiple interactions with the 'external environment (exposome), with nutrition (foodoma) and with pathogens (infectoma), in the context of the genetic variability of the host. Unfortunately, the use of different platforms and original methods developed in-house, as well as the diffusion of structures operating in the sector outside of adequate validation, represents a serious obstacle to the consolidation and large-scale expansion of the results, while promising, so far obtained.

Future challenges in the microbiome and healthcare-related infection control should cover the following objectives:

- To facilitate the clinical application of knowledge in the microbiota by defining typical profiles associated with single individuals, age groups, and groups of pathologies for the characterization of aerobiosis and dysbiotic states of the microbiota in pediatric, adult, and elderly ages and that are related to the development of healthcare-related infections;
- To favor the standardization of diagnostic protocols based on omics technologies (e.g., standardization of sample collection and treatment, optimization of omics procedures and bioinformatic pipelines for interpreting big data), also defining the characteristics of specialists in “microbiology of the microbiota,” able to provide pre-clinical and clinical tools, working closely with other specialists in public health and infectious disease, for the prevention and treatment of healthcare-related infections;
- To define the role of probiotics in improving the balance of the microbiota and their possible effectiveness in maintaining/restoring health and preventing/treating healthcare-related infections, also describing the current state of regulatory aspects and formulating indications for their revision, where deemed useful;
- To contribute to the transferability of the results obtained from research to clinical practice, ensuring safety, application homogeneity, and correspondence to suitably standardized and state-of-the-art procedures;
- To encourage the use in clinical practice of the new diagnostic applications of the microbiota through continuous dialogue with the health governance who are responsible for allowing the use and apply new available technologies.

## Data availability statement

The original contributions presented in the study are included in the article/Supplementary material, further inquiries can be directed to the corresponding author/s.

## Author contributions

PT: conceptualization, methodology, and writing–review and editing. AD: investigation and writing–original draft preparation. LC: supervision and writing–review and editing. All authors have read and approved the final version of the manuscript.

## Funding

This study is part of a Ph.D. project funded by the Italian Ministry of University and Research, NOP Research and Innovation 2014-2020 on innovation and green topics, Ministerial Decree No. 1061 of 10 August 2021.

## Conflict of interest

The authors declare that the research was conducted in the absence of any commercial or financial relationships that could be construed as a potential conflict of interest.

## Publisher's note

All claims expressed in this article are solely those of the authors and do not necessarily represent those of their affiliated organizations, or those of the publisher, the editors and the reviewers. Any product that may be evaluated in this article, or claim that may be made by its manufacturer, is not guaranteed or endorsed by the publisher.
