# Supplementary material for: Human microbiome and microbiota identification for preventing and controlling healthcare-associated infections: A systematic review
Source: Front Public Health. 2022 Dec 1;10:989496. doi: 10.3389/fpubh.2022.989496 (PMC9754121; doi:10.3389/fpubh.2022.989496)
Supplement: Supplementary file 1 [file Table_1.DOCX]

Supplementary Material

## Supplementary Table

| **Reference** | **Healthcare workers’ microbiota and HAIs** | **Patients microbiota and HAIs** | **Healthcare Environment microbiota and HAIs** | **Medical equipment microbiota and HAIs** | **Environmental factors, ecosystem and HAIs** | **Studies of transmission/cleaning and HAIs** | **Resistome and HAIs** | **Number of samples or patients enrolled in the sstudy** | **Care setting** |
| --- | --- | --- | --- | --- | --- | --- | --- | --- | --- |
| Aghdassi SJS et al., 2021 |  |  |  |  | ✔ |  |  | Healthcare associated data from 32118 patients | ✘ |
| Caselli E et al., 2016 |  |  |  |  |  | ✔ | ✔ | 360 samples from healthcare environment | Hospital environment |
| Comar M et al., 2019 |  |  | ✔ |  |  |  | ✔ | 216 samples from healthcare environment | Pediatric clinic, pediatric surgery, pediatric oncology, neonatal intensive and sub-intensive care units, children intensive care unit, surgical rooms and delivery room |
| Costa DM et al., 2019 |  |  | ✔ |  |  | ✔ |  | 57 samples from frequently touched surfaces | Intensive care units |
| Cruz-López F et al., 2020 | ✔ | ✔ | ✔ | ✔ |  |  | ✔ | 11 patients, 35 healthcare workers and 8 patient relatives | Step-down care units |
| Del Campo R et al., 2019 |  |  |  |  |  | ✔ |  | 30 healthy volunteers | ✘ |
| Freedberg DE et al., 2022 |  | ✔ | ✔ |  | ✔ | ✔ |  | 424 samples (304 from patients, 120 from environment) | Intensive care units |
| Gudakova I et al., 2017 |  |  | ✔ |  |  | ✔ |  | 56 samples from environment | Pediatric outpatient facilities |
| Herruzo-Cabrera R et al., 2001 |  |  |  |  |  | ✔ |  | 366 samples from patients | Intensive care units |
| Ke S et al., 2021 |  | ✔ |  |  |  |  |  | 243 patients | ✘ |
| Kelly BJ et al., 2021 |  |  | ✔ |  |  |  |  | 408 samples from healthcare environment | Hospital rooms |
| Lamarche D et al., 2018 |  | ✔ |  |  |  |  |  | 65 samples from patients | Intensive care units |
| Li K et al., 2022 |  |  | ✔ |  | ✔ |  |  | 214 samples from healthcare environment | Intensive care units |
| Lu S et al., 2022 |  | ✔ |  |  |  |  |  | 30 patients and 30 healthy staff members | Intensive care units |
| Lu Y et al., 2022 |  | ✔ |  |  |  |  |  | 300 samples from patients | Neonatal intensive care units |
| Maamar E et al., 2022 |  | ✔ |  |  |  |  | ✔ | 63 patients | Intensive care units |
| Mahjoub H et al., 2022 |  |  |  | ✔ |  |  |  | 33 samples from hospital instrumentation | Ophtalmology clinics |
| McDonald D et al., 2016 |  | ✔ |  |  |  |  |  | 230 samples from patients | Intensive care units |
| Mu S et al., 2022 |  | ✔ |  |  |  |  |  | 34 patients with sepsis, 33 non-septic patients, and 10 healthy individuals | Intensive care units |
| Ogura K et al., 2022 |  | ✔ |  |  |  |  |  | 30 patients | Long-term care hospital |
| Pérez-Fernández T et al., 2020 | ✔ |  | ✔ | ✔ |  | ✔ |  | 201 samples from hospital environment, instuments, equipment, air and physiotherapist. | Physiotherapy and rehabilitation centers |
| Perry-Dow KA et al., 2022 |  |  |  |  |  | ✔ |  | 94 hospital rooms | Rooms of isolated patients with diagnosed infections |
| Ramos T et al., 2015 |  |  |  |  | ✔ |  |  | 12 hospital rooms | Hospital environment |
| Ribeiro LF et al., 2019 |  |  | ✔ |  |  | ✔ |  | 158 samples from healthcare environment | Intensive care units and neonatal intensive care units |
| Schwab F et al., 2020 |  |  |  |  | ✔ |  |  | Healthcare associated data from 1096 patients | Intensive care units |
| Segal E et al., 2022 | ✔ |  |  |  |  |  |  | 7 patients | Intensive care units |
| Sereia AFR et al., 2021 | ✔ | ✔ | ✔ |  | ✔ |  | ✔ | 2160 samples (198 patients, 666 hospital environment, and 216 healthcare workers samples in duplicate) | Hospital environment |
| Sheahan T et al., 2019 |  |  | ✔ |  |  | ✔ |  | 28 samples from vehicles surfaces | Emergency medical services vehicles |
| Shoaei P et al., 2021 | ✔ | ✔ | ✔ | ✔ |  |  | ✔ | 309 samples (189 faecal samples, 29 healthcare workers samples, 51 patients samples, 19 medical device samples, and 21 bed sheets samples) | Tertiary care hospital for injuries related to burn |
| Soffritti I et al., 2022 |  |  |  |  |  | ✔ | ✔ | 152 samples from healthcare environment | Emergency rooms |
| Swanson CS et al., 2022 |  |  |  | ✔ |  |  |  | 20 samples from medical equipment | Medical centre |
| Valeriani F et al., 2016 |  |  |  |  |  | ✔ |  | 48 samples for experimental tests and 83 samples from dental instruments | ✘ |
| Weber KL et al., 2020 |  |  |  |  |  | ✔ |  | 198 samples | ✘ |
| Wiemken TL et al., 2021 |  |  |  |  |  | ✔ |  | 60 samples from healthy adults | ✘ |
| Wu D et al., 2022 |  |  |  |  | ✔ |  | ✔ | 25 samples from hospital environment | ✘ |
| Zakharkina T et al., 2017 |  | ✔ |  |  |  |  |  | 111 samples from patients | Intensive care units |

**Table 1.** Summary of the results obtained from the studies reported in the review. The symbol ✔ indicates the type of result discussed in the article, while the ✘ represents the absence of information.
